# Clinical Cases

**Published:** 1872-09-01

**Authors:** 


### CLINICAL CASES.

FROM THE NOTE BOOK OF N. S. DAVIS, M. D., PROF. PRACTICAL AND CLINICAL MEDICINE IN CHICAGO MEDICAL COLLEGE.

CASE I. *A Sponge Tent in the Uterus Six Weeks.*—Mrs. C—— called at my office for advice in August, 1872. She complained of a constant dull pain in her back and hips, extending often to her thighs, and a constant bloody serous discharge from the vagina, offensive to the smell, and slight general fever. She stated that little more than six weeks previously, a physician who was treating her for some uterine disease, introduced a sponge tent into the mouth and cervix of the womb, and on his attempting to remove it, the string came off and left the sponge behind. And all his efforts to remove it since had failed. On making a simple digital examination per vaginam, I could not satisfy myself that there was anything occupying the mouth of the uterus. The os and whole neck, as far as the finger could reach, were swollen and tender to the touch.

The patient was directed a vaginal wash of sulphate zinc and morphine, dissolved in water, to be used twice per day, and a laxative pill to move the bowels. She returned on the morning of the second day, with all the more important symptoms unchanged. She still insisted that the sponge tent was in the womb, and that the pain in her pelvis and thighs was very severe.

Placing her in a proper position, and introducing the speculum, with a good light the os and neck were brought into full view. The parts were considerably swollen and very red, but not ulcerated. A bloody, serous fluid was oozing from the mouth of the womb so freely as to require to be wiped away with a sponge, and it was very offensive. A probe passed into the os appeared to meet with some obstruction about half an inch from the outer entrance. I then took a pair of medium sized forceps, used for extracting nasal polypi, and carefully crowding the closed blades into the uterine canal and then slightly opening the blades and moving it a little further on endeavored to grasp whatever might be the obstructing body. Finding that something had been actually seized, gentle traction was made, but the instrument soon appeared to slip from its hold and was withdrawn. On examining the blades, however, I found they had brought away a small bit of sponge, a mere shred, but it was sufficient to show that the tent was actually there, as the patient had represented. I re-introduced the forceps, crowding the blades well up to the cavity of the uterus, then opening them with a forward movement, succeeded in grasping the sponge with a firm hold, and by steady, moderate traction, the descent of the uterus being prevented by the blades of the speculum, a sponge tent an inch and a quarter long and nearly half an inch in diameter was brought away. To correct the fœtor of the discharge, as well as to lessen the irritation locally, she was directed to use the following wash with the syringe three times a day.

| | | |
|---|---|---|
| ℞ | Carbolic acid crystals, | 30 grs. |
| | Glycerine, | ℥ ii. |
| | Water, | ℥ vi. |

Mix.

She was also directed to take one teaspoonful of the following mixture before each meal and at bed-time:

| | | |
|---|---|---|
| ℞ | Bromide ammon., | ʒ iv. |
| | Tr. strammonii, | ℥ ss. |
| | Syrup wild cherry, | ℥ ii. |
| | Water, | ℥ iss. |

Mix.

From this time she continued to improve, and in two weeks appeared quite well. The case should teach caution in attaching the ligature to uterine tents.

CASE II. Mrs. S——, aged about 40 years, mother of six or seven children; generally healthy, had a miscarriage in April, 1872, at about the sixth or eighth week of pregnancy. She was in a distant State at the time, and only came under my care recently. She gave the following facts: At the time of the miscarriage the escaped fœtus was observed and fully identified, but I could get from her no satisfactory evidence that the escape of the after-birth had been identified, although her attending physician gave her quite positive assurances that it had. She lost only a moderate amount of blood at the time, and began to get up and look after her household affairs in a few days. She found, however, that there remained an almost constant moderate hemorrhage, with dull, heavy pains in her back, a sense of soreness through the pelvis, and general weakness. These symptoms were pretty uniformly aggravated by walking or any exertion on her feet. In a few weeks the discharge became more dark, and emitted an offensive odor, and often contained little shreds or clots. Of course her strength steadily failed; she had almost constant soreness in the vagina and neck of the bladder, with desire to urinate frequently, in addition to the heavy dragging pain in the back and hips, and soreness across the hypogastric region. She had also a moderate chronic diarrhœa. She was seen often by her family physician who treated her with tonics, astringent vaginal washes, and rest, but made no vaginal examination. When she kept in the recumbent position, the flowing would cease for a day, but was renewed as soon as she made the least exertion, and in spite of her constant use of astringent and deodorizing washes it continued very offensive. In this condition she passed the months of May, June, and July, sitting up a part of almost every day, and sometimes riding out, but gaining no strength, and no exemption from suffering.

About the middle of August a physician made an examination per vaginam, and finding some tough, fleshy substance protruding from the mouth of the uterus, he suspected it to be a polypus, and so informing the lady, she determined to come to Chicago for treatment. Before her arrival here, however, the supposed polypus came away spontaneously, and was described as an irregular, fleshy, and exceedingly offensive mass, in bulk equal to a hen's egg. Considerable hemorrhage had accompanied the escape of the foreign body, and on her arrival a serous bloody discharge still continued, but only moderate in amount. On examination the os-uteri was found large, patulous, tender to the touch, with constant oozing of a dark bloody fluid; and some enlargement and tenderness of the whole cervix and lower part of the body of the uterus. She was kept quiet in the recumbent posture for one week, the vagina washed out night and morning, with the following wash:

| | | |
|---|---|---|
| ℞ | Sulph. zinc | ʒ ii. |
| | Sulph. morph. | 15 grs. |

Mix, divide into 12 powders. One to be dissolved in half a teacupful of water when used.

A solution of persulphate of iron was applied to the os and neck with a sponge, twice at intervals of three days. As the patient had a moderate chronic diarrhœa, with pain in the region of the sigmoid flexure of the colon, she was directed to take one teaspoonful of the following emulsion before each meal and at bed-time:

| | | |
|---|---|---|
| ℞ | Ol. terebinth, | ʒ iii. |
| | Ol. wintergreen, | 30 gtts. |
| | Tinct. opii, | ʒ iv. |
| | Pulv. G. arabic and sugar, aa | ʒ vi. |
| | Rub together and add water, | ℥ iv. |

Mix.

It was thought the turpentine in the prescription would also act favorably in allaying the irritation in the cavity of the uterus. Under this treatment she improved steadily. At the end of one week she began to sit up a part of each day, and in two weeks to ride out. An examination at that time found the cervix and os reduced nearly to the natural size, with only a slight leucorrhœal discharge, and no pain except what was connected with the bowels. She soon after returned to her home.

Several thoughts are suggested by this case. First, the great importance of having all clots or other solid masses passed during a supposed abortion saved until they are carefully examined by the family physician, that he may know with certainty whether the uterus has fully discharged its contents or not. Second, the necessity of a prompt and careful examination of the condition and relations of the uterus in all cases of supposed miscarriages or unusual hemorrhage. A neglect of this has caused mistakes so numerous and important that it should be no longer tolerated. Third, the unusual and interesting fact that the placenta and membranes of a pregnancy of two months were retained after the escape of the fœtus, between four and five months without inducing either pyemia, acute metritis, or fatal hemorrhage.

Case III. *Effects of Alcohol and Opii.*—Mrs. B——, a married lady, who had long been addicted to the excessive use both of alcoholic drinks and opiates, was admitted into the Mercy Hospital early in July, 1872. We learned from her friends that for nearly two months previous to her admission, she had been confined to her room, and mostly to her bed, and almost constantly in a state of mental derangement. She presented a pale, anemic aspect, though fleshy; her skin was cool, pulse small, weak, and quick; respiration natural; the cheeks were of a livid or purplish hue, as if the blood was not perfectly decarbonized; mouth and tongue moist, but the latter coated in the middle and reddish along the edges; the stomach irritable, often rejecting food and drinks; the bowels slightly loose, and urine scanty. Although her attending physician had been several weeks endeavoring to get her gradually off from the use of both alcohol and opium, still she was taking several drinks of the one and several grains of the other daily. Whenever these agents were withheld long enough for their stupefying effects to cease, she became exceedingly restless, moaning, and uttering the most pitiable cries for relief from pains in the lower extremities, and the most horrible mental depression, hallucinations, and sleeplessness. These symptoms would increase until the jactitation, mental anguish and hallucinations, with the feeble pulse and frequent efforts at vomiting, would make the husband and friends think her life in immediate danger, and then they would again give the accustomed anæsthetic or opiate until she became quiet, and she would remain stupid from ten to eighteen hours. The impulse of the heart was feeble, and its systaltic action short and weak, so much so, that we had some fears that fatty degeneration had already commenced in the muscular structure of that organ. As she had already spent two months of utter wretchedness under medical treatment, founded on the policy of trying to withdraw her accustomed poisons gradually, and having no faith in that process, we told the husband and friends plainly and positively that she must not have one drop of any kind of alcoholic drink, or a particle of any preparation of opium, except as we might prescribe, and that her chances of recovery would be greatly increased if they would stay entirely out of her sight and hearing. Having consented to leave her entirely in our hands, they left her only a faithful colored woman as a constant attendant and nurse. We first directed her to have twenty grains of the bromide of potassium every two or three hours, and twenty grains of the hydrate of chloral at bed-time, with beef tea or milk in tablespoonful doses every hour, except when she might be asleep. The house physician was also instructed, in case she became excessively delirious in the first part of the night, to give a hypodermic injection of morphine—one-eighth of a grain—for the first two or three nights.

As was anticipated, the bromide and chloral failed to quiet her in the doses prescribed, and at night the hypodermic injection was given. Under the influence of this she slept heavily all the next morning.

After following the above method of treatment three days without any improvement, the moment she was out from the influence of the morphine or excessive doses of chloral being partially delirious, annoyed by all kinds of visions, noises, and constant restlessness, with the most piteous complaint of pain in the abdomen and lower extremities, we determined to abandon these articles, and as the mucous membrane of both the stomach and rectum was irritable, causing vomiting and frequent inclination to go to stool, she was directed the following mixture:

| | | |
|---|---|---|
| ℞ | Carbolic acid crystals, | 8 grs. |
| | Glycerine | ℥ ss. |
| | Tinct. belladonna, | ℥ ss. |
| | Tinct. digitalis, | ℥ i. |
| | Camph. tinct. opii, | ℥ ii. |
| | Water, | ℥ i. |

Mix. Take one teaspoonful every two hours, until the vomiting and tenesmus ceased, and then lengthen the interval to three hours. In addition she was to have twenty grains of the bromide of potassium at bedtime each night. The same nourishment was continued.

Under this treatment the patient's stomach and bowels steadily improved, but the extreme nervous symptoms were only moderately improved for the first three or four days. Her complaints of distress in the legs and abdomen; her mental wandering and begging for relief and sleep, with the cool extremities and feeble pulse, were well calculated to alarm the friends, and make even the inexperienced physician fear a fatal result, and yield to the entreaty for a *little* of the accustomed stimulants. But having been shown by abundant experience that such yielding only served to protract the condition of the patient indefinitely, while there was no real danger to life, we answered all her entreaties by the positive assurance that she would soon be better, and that under no circumstances could she have a drop of alcoholic drink, or a grain of opium. The moral effect of such assurances in regard to recovery on the one hand, and the utter hopelessness of returning to her former habit, on the other, contributed much towards her final recovery.

We have always found that the sooner such patients could be made to believe not only that they *could live* without those fascinating agents, but also that they positively could not get them on any pretext whatever, the sooner they became tranquil, and the more rapid would be their recovery. The object in giving the digitalis was to impart more steadiness and force to the action of the heart, while the carbolic acid and camphorated tincture of opium should overcome the irritability of the stomach and bowels. After the first three or four days she began steadily to improve; and at the end of four weeks was up, neatly dressed, entirely free from suffering, a fair appetite, and free from delusions, except sometimes the hearing of voices when she slept at night. This, too, will disappear as the brain and nervous structures become more completely renewed by healthy nutrition. To prevent such patients from relapsing into old habits, however, it is of great importance to keep them under observation, with careful attention to the digestive and assimilative functions, for two or three months. They must be aided, mentally and physically, until the morbid impress of the alcohol and opium upon the nerve structures has had time to be eradicated by metamorphosis and renewal of structure.

---

Flies as Transporters of Disease.—A curious and perhaps important discovery has been made recently by M. Kletzinsky, a Viennese professor. Noticing that persons sick with the small-pox were often visited by flies, he placed near an open window of the hospital a saucer filled with glycerine. Soon the flies gathered and were caught like birds with glue. In their endeavors to free themselves the foreign matter which had adhered to them was left in the glycerine, which was at once submitted to the action of a microscope. It was found that this substance, which was chemically pure when offered to the flies, was full of strange cells very like those seen in the vesicles of small-pox, but never on flies. This discovery shows that these insects are not only filthy, but can be very dangerous means of spreading contagious diseases.—*Bost. Med. and Surg. Jour.*, *June* 28, 1872.

Surgical Treatment of Anasarca.—In a severe case of anasarca, Dr. Wolff (*Berlin Klin. Wochenschr.*, No. 141, 1872) introduced into the skin a number of fine canulæ, such as are used for subcutaneous injections, and left them there. Through twenty-five canulæ thus used, twenty quarts of fluid were discharged in three days. The fluid was carried away in elastic tubes into a vessel near the bed. No inflammation of the punctures, nor any other unpleasant results, followed the operation.—*Ibid.*

A Triumph for Lady-Doctors.—The *Medical Times and Gazette*, of May 11th, says: "Our advertising columns to-day contain a novel announcement. The Birmingham and Midland Hospital for Women wants a resident medical officer possessing a medical degree or diploma granted after due examination. Lady-doctors are admissible as candidates. This is the first appointment of the kind which has been opened to ladies in this country."

